# Exploring socio-demographic determinants of obesity in Jordanian women of reproductive age: insights from a nationwide survey

**DOI:** 10.1186/s12889-025-21431-1

**Published:** 2025-01-30

**Authors:** Mahmoud Shaaban Abdelgalil, Sara Hosny El-Farargy, Mohamed Adel Dowidar, Mohamed Abd-ElGawad

**Affiliations:** 1 Faculty of Medicine, Ain-shams University, Cairo, Egypt; 2 Faculty Of Medicine, Benha University, Benha, Egypt; 3 Department of Pharmacy, Sultan Qaboos University Hospital, Muscat, Oman; 4 Faculty of Medicine, Fayoum University, Fayoum, Egypt; 5 Research Insights Arab Network, Cairo, Egypt

**Keywords:** Obesity, Women, Jordan, DHS, JPFHS

## Abstract

**Background:**

In Jordan, obesity has emerged as a significant public health concern, particularly among females, with a prevalence of 43.1%. This rising trend, exacerbates the burden of non-communicable diseases and places increasing strain on the healthcare system.

**Aim:**

We aimed to explore the predictors associated with obesity among adult ever-married Jordanian women aged 20–49 years based on the Jordanian Population and Family Health Survey (JPFHS).

**Method:**

Our study analyzed data from the JPFHS conducted in 2017-18, which initially included 14,689 ever-married women. We performed multivariable analyses to determine the socio-demographic predictors of obesity among these women.

**Result:**

We included 4,339 Jordanian women in our study, of whom 2,189 were classified as obese and 2,150 had a normal body mass index (BMI). Multivariable analysis indicated that increasing age and living in Tafilahwere significantly associated with higher odds of developing obesity (*p* < 0.05). Conversely, factors such as being in the wealthiest category, residing in Ma’an and Aqaba, and smoking every day were significantly linked to reduced odds of obesity (*p* < 0.05). Additionally, no significant associations were found between obesity development and variables such as the type of place of residence, educational level, frequency of reading newspapers or magazines, radio listening, television watching, or internet use in the past month (*p* > 0.05).

**Conclusion:**

Appropriate and targeted interventions should be developed for Jordanian women to address obesity and its related health issues. Policymakers should adopt a multilevel approach that focuses on high-risk subgroups, including older women, and those living in Tafilh. Efforts should be made to raise awareness and provide preventative measures tailored to these groups to effectively reduce obesity and its associated complications.

## Introduction

Obesity has become a critical public health challenge globally, particularly in low- and middle-income countries undergoing rapid socioeconomic transitions [1]. The World Health Organization defines obesity as an excessive fat accumulation that poses significant health risks [2]. Over recent decades, global obesity rates have escalated dramatically, with the Middle East, including Jordan, experiencing a notable increase [3].

Current estimates indicate that approximately 2 billion adults worldwide are overweight, with 677.6 million classified as obese [4]. Women represent a significant proportion of this group, comprising 393.5 million of the global obese population [5]. Obesity in women is associated with numerous adverse health outcomes, including increased risks of cardiovascular diseases, type 2 diabetes, and various malignancies, such as breast, ovarian, and endometrial cancers [6–8]. These conditions contribute to premature mortality, reduced productivity, economic burdens on healthcare systems, and psychological distress [9, 10]. The COVID-19 pandemic further underscored the vulnerability of obese individuals, demonstrating an elevated risk of severe outcomes, including hospitalization and mortality. A recent meta-analysis of 75 international studies found that obese adults face a 113% higher risk of hospitalization, a 74% higher risk of ICU admission, and a 48% increased risk of death compared to those with normal weight [11]. This escalating obesity crisis imposes a significant strain on healthcare systems and carries a global economic burden of approximately $2 trillion annually [12]. Additionally, maternal obesity is associated with a higher likelihood of obesity in offspring, perpetuating an intergenerational cycle of obesity [13, 14].

While overweight and obesity were once considered problems exclusive to high-income countries, they have become global issues, impacting low- and middle-income nations as well, including those in the Eastern Mediterranean Region (EMR) [6, 15]. Within the EMR, which includes Jordan, obesity rates are alarmingly high, significantly contributing to the burden of non-communicable diseases. In Jordan, the rising prevalence of obesity among adults presents a significant challenge to the healthcare system [3]. Obesity rates are notably higher among females, at 43.1%, compared to 28.2% among males [16]. Over the past two decades, this prevalence has increased at an average annual rate of 1.38% [16]. Key factors contributing to this trend include dietary shifts, sedentary lifestyles, urbanization, marital status, and cultural norms [3].

In Jordan, reproductive health indicators, such as fertility rates, breastfeeding practices, and delivery methods, are influenced by both traditional cultural norms and the forces of globalization. The total fertility rate was 2.7 births per woman in 2017, showing a decline from previous decades [17]. While over 90% of children are breastfed at some point, only 67% are breastfed within the first hour of life, and nearly half (43%) receive prelacteal feeds, despite health guidelines advising against them. The World Health Organization recommends exclusive breastfeeding for the first six months, but only 26% of children under six months in Jordan follow this practice [17]. Cesarean section rates have also increased, with over 26% of births delivered by C-section in 2017 [17]. Culturally, traditional diets in Jordan are rich in grains, legumes, and fresh produce, but globalization has shifted dietary habits towards processed foods, fast foods, and sugary beverages, contributing to the rising prevalence of obesity and related non-communicable diseases. Urbanization, longer working hours, and increased food imports—accounting for around 70% of the country’s staples—have further changed food consumption patterns, affecting both food affordability and accessibility [18].

Musaiger et al.‘s systematic review of obesity in the EMR identified several key factors that contribute to the increased risk of obesity in the region. These factors include dietary shifts, physical inactivity, urbanization, marital status, shorter breastfeeding durations, frequent snacking, skipping breakfast, high consumption of sugary drinks, increased dining out, prolonged television viewing, aggressive marketing of high-fat foods, stunting, body image perceptions, cultural influences, and food subsidy policies [19].Another study using data from the Jordan Population and Family Health Survey (JPFHS) 2009 reported that factors such as age, residence in the southern regions of Jordan, early marriage, parity, wealth status, and smoking are associated with obesity over time [20].

Despite existing research, there is a lack of recent evidence on the socioeconomic, behavioral, and reproductive factors linked to obesity. This study aims to investigate the socio-demographic correlates of obesity among adult ever-married women in Jordan by utilizing updated datasets from the JPFHS 2017-18.

## Methods

### Data source

This study utilized data from the JPFHS conducted in 2017-18. The survey included a nationally representative sample of women aged 15–49 years across Jordan’s 12 governorates. Anthropometric measurements, including body mass index (BMI), were collected to assess the nutritional status of the participants.

### Inclusion criteria

The study focused on adult ever-married women aged 20–49 years who had complete BMI data. Women with a BMI measured according to the Centers for Disease Control and Prevention guidelines were included [21]. Specifically, the analysis included women with normal BMI (18.5–24.9) and those classified as obese (BMI > 30).

### Exclusion criteria

Women who had missing BMI data, those classified as underweight (BMI < 18.5), overweight (BMI 25–29.9), or women aged below 20 years were excluded from the analysis, as the study specifically focused on ever-married women. Women with underweight (BMI < 18.5) or overweight (BMI 25–29.9) were excluded to ensure a targeted analysis of the socio-demographic and behavioral factors associated with normal and obesity-related BMI categories.

### Included variables

In our cross-sectional study, we analyzed a variety of sociodemographic and behavioral variables to assess their association with obesity among Jordanian women of reproductive age. Age was categorized into six 5-year groups: 20–24, 25–29, 30–34, 35–39, 40–44, and 45–49 years. The type of place of residence was classified as either urban or rural. Educational level was divided into four categories: no education, primary, secondary, and higher. The wealth index was divided into five categories—poorest, poorer, middle, richer, and richest—based on the DHS wealth index methodology, which classifies households according to asset data [22]. Regional analysis was conducted across three major regions: Central (Amman, Balqa, Zarqa, Madaba), Northern (Irbid, Mafraq, Jerash, Aljoun), and Southern (Karak, Tafilah, Ma’an, Aqaba).

We also examined the frequency of media consumption, including reading newspapers or magazines, listening to the radio, and watching television, each categorized as not at all, less than once a week, or at least once a week. Household characteristics included whether the household owned a car or truck (yes/no) and the type of cooking fuel used, categorized as electricity, natural gas, kerosene, coal/lignite, or no food cooked in-house. Internet usage over the past month was assessed as not at all, less than once a week, at least once a week, or almost every day. Finally, we evaluated the current contraceptive method used by participants, including options such as not using any method, pill, IUD, injections, male condom, female sterilization, male sterilization, periodic abstinence, withdrawal, implants/Norplant, and lactational amenorrhea (LAM).

### Statistical analysis

Data were analyzed using SPSS version 24. We used a weighted count for the analysis based on DHS recommendations [23]. The sample weight was an eight-digit variable with six implied decimal places, and the weighting factor was divided by 1,000,000 for application. Descriptive statistics were used to summarize the characteristics of the study population, with results reported as frequencies and percentages. Multivariable logistic regression analyses were performed to identify predictors of obesity. The results were presented as adjusted odds ratios (AORs) with 95% confidence intervals (CIs). A p-value of less than 0.05 was considered statistically significant.

## Results

Data were collected from 4,339 Jordanian women of reproductive age (20–49 years). Of these participants, 2,150 were classified as obese, while 2,189 had a normal BMI (Table 1).

**Table 1:** Characteristics of the included women according to BMI

Variables		BMI	
		Normal (BMI = 18.5: 24.9) *N* = 2150	Obese (BMI ≥ 30) *N* = 2189
		Count N %	Count N %
Age in 5-year groups	20–24	343 (16.7%)	116 (5.3%)
	25–29	509 (24.8%)	180 (8.3%)
	30–34	442 (21.5%)	312 (14.3%)
	35–39	306 (14.9%)	404 (18.6%)
	40–44	256 (12.5%)	559 (25.7%)
	45–49	197 (9.6%)	603 (27.7%)
Type of place of residence	Urban	1927 (89.6%)	1930 (88.2%)
	Rural	223 (10.4%)	259 (11.8%)
Highest educational level	No education	52 (2.4%)	59 (2.7%)
	Primary	108 (5.0%)	180 (8.2%)
	Secondary	1173 (54.6%)	1328 (60.7%)
	Higher	816 (38.0%)	622 (28.4%)
Wealth index combined	Poorest	448 (20.8%)	481 (22.0%)
	Poorer	392 (18.2%)	476 (21.7%)
	Middle	463 (21.5%)	470 (21.5%)
	Richer	416 (19.4%)	457 (20.9%)
	Richest	431 (20.0%)	305 (13.9%)
Region	Central Region		
	Amman	860 (40.0%)	817 (37.3%)
	Balqa	130 (6.0%)	104 (4.8%)
	Zarqa	270 (12.6%)	348 (15.9%)
	Madaba	44 (2.0%)	57 (2.6%)
	North Region		
	Irbid	369 (17.1%)	418 (19.1%)
	Mafraq	130 (6.0%)	159 (7.3%)
	Jerash	63 (2.9%)	66 (3.0%)
	Ajloun	50 (2.3%)	48 (2.2%)
	South Region		
	Karak	80 (3.7%)	75 (3.4%)
	Tafilah	21 (1.0%)	47 (2.1%)
	Ma’an	53 (2.5%)	23 (1.0%)
	Aquaba	81 (3.8%)	26 (1.2%)
Frequency of reading newspaper or magazine	Not at all	1240 (57.7%)	1323 (60.4%)
	Less than once a week	460 (21.4%)	430 (19.6%)
	At least once a week	450 (20.9%)	436 (19.9%)
Frequency of listening to radio	Not at all	1132 (52.6%)	1179 (53.9%)
	Less than once a week	502 (23.3%)	504 (23.0%)
	At least once a week	517 (24.0%)	506 (23.1%)
Frequency of watching television	Not at all	196 (9.1%)	190 (8.7%)
	Less than once a week	355 (16.5%)	357 (16.3%)
	At least once a week	1599 (74.4%)	1641 (75.0%)
Frequency of using the Internet last month	Not at all	425 (19.7%)	598 (27.3%)
	Less than once a week	49 (2.3%)	61 (2.8%)
	At least once a week	175 (8.1%)	186 (8.5%)
	Almost every day	1502 (69.8%)	1344 (61.4%)
Frequency smokes cigarettes	Do not smoke	1941 (90.3%)	2032 (92.9%)
	Every day	139 (6.5%)	110 (5.0%)
	Some days	70 (3.3%)	46 (2.1%)

The analysis revealed that the highest proportion of obese women was in the 40–49 age group, accounting for 53.4% of the total obese population. In contrast, 46.3% of women with a normal BMI were between 25 and 34 age groups. Regarding residence, 88.2% of women with a normal BMI and 89.6% of obese women lived in urban areas. Among women with a normal BMI, over half had completed secondary education, while 5.0% had completed primary education, 38% had higher education, and 2.4% had no formal education. In the obese group, 60.7% had secondary education, 8.2% had primary education, 28.4% had higher education, and 2.7% had no formal education.

**Fig. 1:**
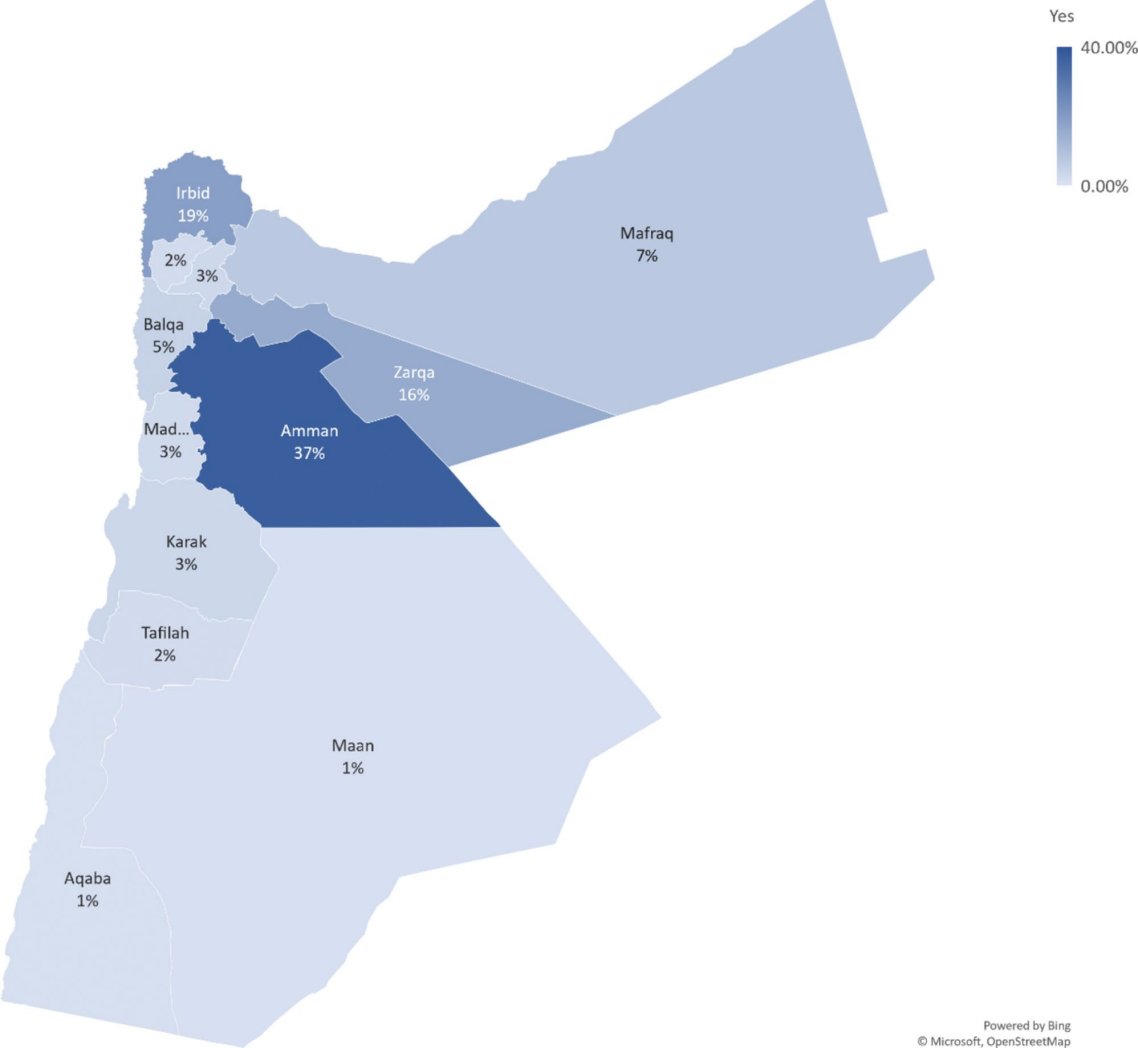
Geographical Distribution of Obesity Among Jordanian Women by Governorate

Obesity was least prevalent among women in the richest wealth quintile, affecting 13.9% of women in this group. In comparison, obesity rates were higher in the richer (20.9%), middle (21.5%), poorer (21.7%), and poorest (22.0%) quintiles. Conversely, the distribution of women with a normal BMI was relatively similar across wealth categories: poorest (20.8%), poorer (18.2%), middle (21.5%), richer (19.4%), and richest (20.0%). The distribution of participants varied across regions. The highest proportions were seen in Amman, with 40% of normal BMI and 37.3% of obese women Fig. 1.

In terms of media consumption, 57.7% of women with a normal BMI and 60.4% of obese women did not read newspapers or magazines at all. Among those who did, 21.4% of women with a normal BMI and 19.6% of obese women read newspapers or magazines less than once a week, while 20.9% of women with a normal BMI and 19.9% of obese women read them at least once a week.

Similarly, when it came to radio listening, 52.6% of women with a normal BMI and 53.9% of obese women reported not listening at all. Of those who did, 23.3% of women with a normal BMI and 23.0% of obese women listened less than once a week, while 24.0% of women with a normal BMI and 23.1% of obese women listened at least once a week.

In terms of television watching, our results showed that most women with a normal BMI (74.4%) and those with obesity (75.0%) watched television at least once a week. Regarding Internet usage over the past month, most women with a normal BMI (69.8%) and those with obesity (61.4%) accessed it almost every day. When it comes to cigarette smoking, most women with a normal BMI (90.3%) and those with obesity (92.9%) are non-smokers.

In our multivariate analysis (Table 2), a clear trend of increasing obesity odds was observed with advancing age. Compared to the reference group (ages 20–24), the adjusted odds ratios (AOR) for obesity were significantly higher in the older age groups: 30–34 years (AOR: 2.36; 95% CI: 1.62–3.44; *p* < 0.001), 35–39 years (AOR: 4.72; 95% CI: 3.14–7.10; *p* < 0.001), 40–44 years (AOR: 8.12; 95% CI: 5.42–12.15; *p* < 0.001), and 45–49 years (AOR: 11.78; 95% CI: 7.96–17.44; *p* < 0.001). However, the age group 25–29 years (AOR: 1.13; 95% CI: 0.77–1.66; *p* = 0.534) did not show a significant association with obesity.

**Table 2:** Predictors of women’s obesity

Parameter Estimates							
Variables		B	Standard Error	AOR	95% Confidence Interval for AOR		P-value
					Lower	Upper	
Age in 5-year groups	20–24	Reference					
	25–29	0.121	0.195	1.129	0.770	1.655	0.534
	30–34	0.859	0.192	2.360	1.620	3.440	< 0.001
	35–39	1.552	0.208	4.723	3.140	7.103	< 0.001
	40–44	2.094	0.206	8.117	5.422	12.151	< 0.001
	45–49	2.466	0.200	11.781	7.956	17.444	< 0.001
Highest educational level	No education	Reference					
	Primary	0.532	0.334	1.702	0.883	3.279	0.112
	Secondary	0.330	0.269	1.391	0.820	2.359	0.220
	Higher	0.134	0.287	1.143	0.651	2.008	0.642
Type of place of residence	Urban	Reference					
	Rural	0.051	0.149	1.053	0.786	1.410	0.730
Wealth index combined	Poorest	Reference					
	Poorer	0.143	0.152	1.153	0.856	1.555	0.349
	Middle	-0.125	0.158	0.883	0.648	1.204	0.430
	Richer	-0.235	0.178	0.790	0.558	1.120	0.186
	Richest	-0.818	0.215	0.441	0.289	0.673	< 0.001
Region	Amman	Reference					
	Balqa	-0.306	0.208	0.736	0.489	1.108	0.142
	Zarqa	0.225	0.178	1.252	0.883	1.776	0.207
	Madaba	0.245	0.180	1.277	0.897	1.818	0.174
	Irbid	0.110	0.171	1.116	0.797	1.562	0.523
	Mafraq	0.145	0.179	1.156	0.814	1.642	0.417
	Jerash	-0.094	0.263	0.910	0.543	1.524	0.720
	Aljoun	-0.125	0.236	0.883	0.555	1.404	0.598
	Karak	-0.159	0.219	0.853	0.555	1.311	0.468
	Tafilah	0.810	0.206	2.248	1.501	3.366	< 0.001
	Ma’an	-0.958	0.231	0.384	0.244	0.604	< 0.001
	Aquaba	-1.250	0.228	0.287	0.183	0.449	< 0.001
Frequency of reading newspaper or magazine	Not at all	Reference					
	Less than once a week	-0.182	0.148	0.834	0.624	1.114	0.219
	At least once a week	-0.171	0.161	0.843	0.615	1.155	0.288
Frequency of listening to radio	Not at all	Reference					
	Less than once a week	0.116	0.136	1.123	0.861	1.466	0.391
	At least once a week	0.111	0.159	1.118	0.818	1.527	0.484
Frequency of watching television	Not at all	Reference					
	Less than once a week	0.131	0.222	1.140	0.738	1.761	0.554
	At least once a week	0.153	0.181	1.166	0.818	1.663	0.396
Frequency of using the Internet last month	Not at all	Reference					
	Less than once a week	0.188	0.318	1.206	0.647	2.250	0.555
	At least once a week	-0.087	0.180	0.917	0.644	1.306	0.631
	Almost every day	-0.055	0.140	0.947	0.720	1.245	0.695
Frequency smokes cigarettes	do not smoke	Reference					
	some days	-0.252	0.396	0.778	0.358	1.690	0.525
	every day	-0.503	0.230	0.605	0.385	0.951	**0.029**

Women with primary education had an AOR of 1.70 (95% CI: 0.88–3.28; *p* = 0.112), those with secondary education had an AOR of 1.39 (95% CI: 0.82–2.36; *p* = 0.220), and higher education was associated with an AOR of 1.14 (95% CI: 0.65–2.01; *p* = 0.642).Also, Rural residency did not show a significant relationship with obesity (AOR: 1.05; 95% CI: 0.79–1.41; *p* = 0.73).

Wealth quintiles showed varied impacts on obesity risk. Participants in the poorer (AOR: 1.15; 95% CI: 0.86–1.56; *p* = 0.349), middle (AOR: 0.88; 95% CI: 0.65–1.20; *p* = 0.430), and richer (AOR: 0.79; 95% CI: 0.56–1.12; *p* = 0.186) groups had no significant difference in obesity odds compared to the poorest quintile. However, those in the richest quintile had a significantly lower likelihood of obesity (AOR: 0.44; 95% CI: 0.29–0.67; *p* < 0.001).

Regional differences in obesity were notable. Tafilah had a significantly higher likelihood of obesity compared to Amman (AOR: 2.25; 95% CI: 1.50–3.37; *p* < 0.001), while Ma’an (AOR: 0.39; 95% CI: 0.24–0.60; *p* < 0.001) and Aqaba (AOR: 0.29; 95% CI: 0.18–0.45; *p* < 0.001) showed significantly lower odds.

In our analysis of smoking and obesity, we found that individuals who smoked every day were significantly less likely to be obese, with an odds ratio of 0.61 (95% confidence interval: 0.39–0.95; *p* = 0.029). Conversely, individuals who smoked only on some days did not show a significant association with obesity, as indicated by an odds ratio of 0.78 (95% confidence interval: 0.36–1.69; *p* = 0.525).

Our analysis found no significant association between obesity and the frequency of watching television, listening to the radio, reading newspapers or magazines, and using the Internet last month.

## Discussion

Our study is the first to identify obesity risk factors among adult women in Jordan using nationally representative data. The analysis revealed a strong correlation between age and obesity, with older women being significantly more affected. Specifically, women aged 44–49 were 11.7 times more likely to be obese compared to those aged 20–24. These findings align with studies by Al Nsour et al. in Jordan 2008 [20], Rawal et al. in Nepal 2018 [24], Mbochi et al. in Nairobi 2012 [25], and Mukora et al. in Zimbabwe 2019 [26], which also reported higher obesity rates among older women. This trend may be attributed to the hormonal changes that occur with aging. As women approach menopause, a decline in estrogen and an increase in circulating androgens lead to significant shifts in body composition. These hormonal changes contribute to muscle loss, increased abdominal fat, and alterations in body shape. When combined with a sedentary lifestyle, these factors reduce overall energy expenditure and basal metabolic rate, further elevating the risk of obesity in this demographic [27, 28].

Moreover, as women age, a decline in physical function often occurs, partly due to significant changes in body composition, such as sarcopenia—the age-related reduction in skeletal muscle mass. This decline in muscle mass can lead to slower gait speed, impaired balance and coordination, reduced bone mineral density, and a diminished quality of life. Physical activity plays a crucial role in counteracting these physiological declines associated with aging [29]. Therefore, maintaining adequate physical activity levels can enhance longevity and reduce the risk of metabolic and other chronic diseases.

During menopause, women often experience significant lifestyle changes that can affect their quality of life. Since women may live through menopause for more than a third of their lives, maintaining good health during this period is essential. Adopting a healthy diet and staying physically active are key factors in promoting health and well-being during menopause. Therefore, evaluating the adherence to healthy lifestyle practices, such as diet and exercise, among menopausal women is crucial [30].

A study by Alnjadat et al. [30] assessed adherence to healthy diets and physical activity among menopausal women in Jordan and identified the factors influencing these behaviors. The results showed that menopausal women in Jordan generally exhibited moderate adherence to both healthy eating and physical activity. Age, number of children, and education level were significant predictors of adherence to these practices. Specifically, women with two children and those with primary or secondary education were more likely to maintain a healthy lifestyle [30].

Additionally, a study by Assaf et al. [31] examined the influence of employment status on the quality of life for menopausal women. Working women reported a higher quality of life compared to retired women, likely due to the social engagement and financial independence that employment offers. On the other hand, retirement can lead to reduced social interaction and a sense of loss of purpose, negatively impacting both mental and physical health. Therefore, remaining employed or engaging in community activities during menopause can be beneficial for women’s overall health and well-being [31]. It is recommended that menopausal women maintain a healthy diet, engage in regular physical activity, and stay socially and economically active to help prevent obesity and improve overall well-being.

The analysis of the wealth index and its association with obesity among women yielded noteworthy findings. Women categorized as “Richest” were significantly less likely to be obese compared to those in the “Poorest” category. However, no statistically significant differences in obesity odds were observed among the “Poorer,” “Middle,” and “Richer” categories when compared to the “Poorest” group. Our results are consistent with the study by Al Nsour et al. 2008 [20]. conducted in Jordan. However, they contrast with findings from Rawal et al. in Nepal 2022 [1], Mukora et al. in Zimbabwe 2020 [3], and El-Qushayri et al. in Egypt 2023 [9], which reported a higher risk of obesity with increasing wealth. One possible explanation for our results is that extreme wealth may confer protection against obesity, potentially due to better access to healthier food, healthcare, and lifestyle choices. Tara Templin’s research suggests that, in high-income countries, obesity is more prevalent among poorer populations, reflecting a shift in obesity patterns as countries develop [32]. While higher wealth often improves dietary choices and health outcomes, it can also lead to weight gain due to lifestyle changes associated with increased affluence, particularly among women [33, 34]. This underscores the need for public health strategies that account for varying socioeconomic dynamics to effectively address obesity [32, 34].

The analysis of obesity odds across different regions reveals significant variations. In the Central and Northern regions, there were no notable differences in obesity odds. However, In the Southern region, there was variability in obesity rates between different areas. Specifically, living in Tafilah was associated with an increased likelihood of obesity, whereas residing in Ma’an and Aqaba was linked to a decreased likelihood of obesity. This finding aligns with the study by Al Nsour et al. conducted in Jordan in 2009 which reported a significant increase in obesity odds across both northern and southern regions of Jordan, without specifying the areas associated with this risk [20].

These regional differences may be attributed to socioeconomic factors, with the Central regions generally having a higher socioeconomic status compared to the Northern and Southern regions [16, 35]. Variations in obesity prevalence by region may reflect the impact of education and socioeconomic status on health. Higher educational attainment and socioeconomic status are often associated with greater health awareness and more positive attitudes toward healthy lifestyles, in contrast to those with lower levels of education and socioeconomic status [36]. Additionally, fertility rates may influence regional differences, as in Tafilah, where a higher fertility rate of 3.0 is associated with larger family sizes. This can limit the time and resources available for women to focus on self-care and healthy behaviors. In contrast, in Ma’an and Aqaba, where fertility rates are lower at 2.6, women may face less pressure from larger families, potentially allowing more opportunities for health-promoting activities. We recommend conducting further research to gain a deeper understanding of the variations in obesity prevalence across different regions.

The analysis of smoking frequency reveals an interesting association. Notably, daily smoking among women is significantly associated with lower obesity rates, aligning with previous findings by Al Nsour et al. 2009 [20], Watanabe et al. 2016 [37], and Dare et al. 2015 [38]. According to the 2017/18 DHS data for Jordan, 12% of ever-married women aged 15–49 smoke tobacco [17].

The relationship between smoking and obesity is complex and influenced by multiple factors. While current smokers tend to have a lower obesity risk—likely due to nicotine’s metabolic effects and appetite suppression—former smokers often experience increased central fat accumulation and higher obesity rates [39]. Smokers tend to have higher levels of central obesity compared to non-smokers, as evidenced by increased waist circumferences and waist-to-hip ratios. This relationship remains significant even after adjusting for body mass index, indicating that smoking primarily influences fat distribution rather than total body weight [40, 41].

Watanabe et al. observed that the prevalence of obesity often rises with the number of cigarettes smoked daily and cumulative pack-years, though not necessarily with the duration of smoking [37]. Thus, while smoking may be linked to reduced obesity risk, it also brings substantial health risks, including higher rates of central adiposity, cardiovascular disease, and cancers [39, 42].

Importantly, the evidence does not support the belief that smoking protects against weight gain. Highlighting this in educational programs may be an effective strategy for discouraging smoking initiation among youth.

Trends in nutritional status in Jordan have become significant predictors of obesity, driven by a rapid nutrition transition. This shift from undernutrition to overnutrition, characterized by high fat, sugar, and refined carbohydrate consumption, has led to rising obesity rates, especially among women. Obesity is notably higher among women with lower education levels (50%). The average caloric intake has increased, with fats contributing 38% of total energy, exceeding recommended levels, while fiber intake remains insufficient. Urbanization and socioeconomic changes, including high poverty rates, have further contributed to unhealthy dietary patterns. These trends underscore the need for interventions promoting healthier diets and physical activity to address the obesity epidemic [43, 44].

### Strength and limitation

The key strength of our study lies in the use of nationally representative data to identify predictors of obesity among Jordanian women of reproductive age. Additionally, the JDHS data collection tool is standardized and validated, minimizing the potential for bias and error compared to smaller studies.

However, our study has several limitations that warrant further investigation in future research. Firstly, the cross-sectional design of the study limits our ability to establish causality between obesity and its associated risk factors or complications. Secondly, the survey only included participants up to 49 years old, highlighting the need for future studies to include older age groups. Thirdly, the survey data did not account for comorbidities, which are crucial in the development of obesity. Lastly, since only ever-married women were eligible for the study, we were unable to include marital status as a predictor of obesity.

### Recommendation

Based on study findings, several recommendations can be made to address obesity among Jordanian women. Given the strong correlation between age and obesity, targeted interventions should be developed for older women, focusing on promoting healthy lifestyle choices and regular physical activity. Efforts should also be made to bridge the disparity between wealth categories by ensuring that all socioeconomic groups have access to resources that promote healthy weight management. The regional variations in obesity odds, particularly in the Southern region, highlight the importance of region-specific public health strategies that address local cultural and socioeconomic factors. Future research should examine the relationship between smoking and obesity in Jordan, which could be more finely tuned by examining the influences of the number of cigarettes smoked daily, cumulative pack-years, and years of smoking on the prevalence of obesity among women.

## Conclusion

In conclusion, older women and those living in Tafilah are associated with higher obesity rates among Jordanian women. Conversely, wealthier women and those residing in Ma’an and Aqaba are linked to lower obesity rates. Implementing targeted interventions that focus on these specific demographics and regions could help reduce obesity and its related health risks.

## Data Availability

Data is available upon request from ICF International's website (https://dhsprogram.com/data/available-datasets.cfm).

## Abbreviations

AOR: Adjusted odds ratio
